# B cell activating factor in juvenile onset systemic lupus erythematosus, looking beyond the B cell

**DOI:** 10.1186/1546-0096-12-S1-P112

**Published:** 2014-09-17

**Authors:** Ella Richards, Angela Midgely, Thomas Morgan, Michael Beresford

**Affiliations:** 1 Institute of Child Health, Univeristy of Liverpool, Liverpool, UK

## Introduction

Increased B cell activating factor (BAFF) in the serum of juvenile-onset systemic lupus erythematosus (JSLE) patients is thought to be key to the survival of auto-reactive B cells. BAFF signals through three receptors, BAFF-R, BCMA and TACI, activating the NF-κB pathway. Belimumab is a human monoclonal antibody against BAFF that has recently become the first drug licensed for the treatment of SLE in 50 years. In order to investigate the potential effect of BAFF inhibition on the wider immune system, receptor expression was investigated in another SLE pathogenic cell, the T lymphocyte.

## Objectives

To determine T cell expression of the BAFF-R and BCMA receptors and the effect of BAFF on cell survival in JSLE patients compared to healthy paediatric controls.

## Methods

Lymphocytes were isolated from JSLE patients and healthy paediatric controls. Cells were dual immunostained with flurochrome conjugated monoclonal antibodies for BAFF receptors, BAFF-R or BCMA (FITC labelled) as well as the T cell marker CD3 (PECy5 labelled) allowing detection of T cells expressing the receptor to be identified by flow cytometry (n=8). Survival of T cells was assessed by quantifying the degree of apoptosis taking place using an Annexin V stain after 4 hours incubation with recombinant human BAFF (rhBAFF); receptor expression was analysed as stated previously (n=5). Results expressed as: mean+/-SEM. Statistical significance was taken when p values were <0.05.

## Results

The total lymphocyte population was found to express both BAFF-R and BCMA receptors similarly in JSLE patients (BAFF-R 14.8%+/-3.15; BCMA 4.98+/-0.86) and controls (BAFFR 8.2%+/-1.6; BCMA 6.07+/-0.87). T cells also displayed similar expression (JSLE: BAFF-R 3.37%+/-0.42; BCMA: 1.91+/-0.31; Controls: BAFF-R, 3.27%+/-0.82; BCMA, 2.34+/-0.96) with BAFF-R receptor expressed more frequently than BCMA in both cohorts. Importantly, incubation with rhBAFF led to an increase in the survival of T cells when analysing both JSLE patients and controls together (n=9 pre treatment 25%+/-3.1; rhBAFF treated, 19.7+/-2.7; p=0.008), and separately (JSLE: pre treatment, 31.04%+/-2.34; rhBAFF treated, 25.03%+/-1.13, p=0.043; Control: pre-treatment, 17.59%+/-4.27; rhBAFF treated, 12.99%+/-4.24, p=0.068). rhBAFF also significantly down-regulated BAFF-R expression in lymphocytes of both groups (n=9) Pre-treated; 11.51%+/-2.00; rhBAFF treated: 5.02%+/-0.907) . Finally a positive correlation with the disease activity score SLEDAI was observed with JSLE patient BCMA receptor expression on both lymphocytes (correlation coefficient r^2^=0.6 p=0.024) and T cells (r^2^=0.67; p=0.013).

## Conclusion

This study has demonstrated that T cells from paediatric patients express BAFF receptors as previously noted in adults. Interestingly both BAFF-R and BCMA receptors were detected. Whether by direct signalling or an indirect mechanism, BAFF has been shown to reduce apoptosis of T cells in both JSLE patients and healthy controls. This observation challenges the notion that T cells exclusively express BAFF-R highlighting that BCMA expression on T cells could be unique to the immature immune system. Importantly, these data indicate that this ‘B cell survival factor’ is also capable of influencing the survival of T cells *in vitro*. Aberrant T cell homeostasis is a pathogenic feature of SLE. To understand how increased T cell survival may affect the disease, it is important to understand receptor expression on different T cell subsets, including the inflammatory Th17 and anti-inflammatory Tregs. Allowing a clearer picture of how BAFF inhibition may affect the wider immune system.

## Disclosure of interest

None declared.

